# Crosslinking of Bovine Gelatin Gels by Genipin Revisited Using Ferrule-Top Micro-Indentation

**DOI:** 10.3390/gels9020149

**Published:** 2023-02-10

**Authors:** Vincent Ball

**Affiliations:** 1 Faculté de Chirurgie Dentaire, Université de Strasbourg, 8 rue Sainte Elizabeth, 67000 Strasbourg, France; vball@unistra.fr; 2 Institut National de la Santé et de la Recherche Médicale, Unité Mixte de Recherche, 1 Rue Eugène Boeckel, CEDEX, 67084 Strasbourg, France

**Keywords:** ferrule-top micro-indentation, rheology, gelatin: crosslinking with genipin

## Abstract

(1) Background: Gelatin is widely used in food science, bioengineering, and as a sealant. However, for most of those applications, the mechanical properties of gelatin gels need to be improved by means of physical or chemical crosslinking. Among the used chemical agents, genipin allows low cytotoxicity in addition to improved Young’s modulus. However, the mechanical properties of gelatin–genipin gels have only been investigated at the macroscale, and there is no knowledge of the influence of the genipin concentration on the surface homogeneity of Young’s modulus. (2) Methods: To this aim, the influence of genipin concentration on Young’s modulus of gelatin gels was investigated by means of ferrule-top micro-indentation. The data were compared with storage moduli obtained by shear rheology data. (3) Results: Ferrule-top indentation measurements allowed us to show that Young’s moduli of gelatin–genipin gels increase up to a plateau value after approximately 12 mg/mL in genipin and 4 h of crosslinking. Young’s moduli distribute with high homogeneity over 80 µm × 80 µm surface areas and are consistent with the storage moduli obtained by shear rheology. (4) Conclusions: It has been shown that ferrule-top indentation data fitted with the Hertz model yield Young’s moduli of gelatin–genipin gels which are consistent with the storage moduli obtained by characterization at the macroscale using shear rheometry. In addition, Young’s moduli are homogenously distributed (with some irregularities at the highest genipin concentrations) and can be increased by two orders of magnitude with respect to the uncrosslinked gel.

## 1. Introduction

Among hydrogels finding a common use in food science and biomaterials science, gelatin gels are of paramount importance, partly due to the great availability of proteins among different animal sources. Their gelation and properties depend markedly on the animal source [1]. Those gels are obtained by cooling a hot gelatin sol above a critical concentration either under a temperature gradient or by quenching the gel at a constant temperature below the critical gelation temperature. In both cooling protocols, the material never reaches an equilibrium state [2,3], but the fraction of triple helices slightly increases to become closer to that of the collagen used to produce gelatin. Gelatin has been blended with other polymers, for instance, polyurethane, to yield hydrogels used for bioprinting [4]. Even if the mechanical properties of gelatin gels depend strongly on the animal from which gelatin has been obtained [1], those gels are pretty weak and dissolve spontaneously at the temperature of the human body which is above the gel–sol transition of the gelatin-based gels. For this reason, many attempts have been made to transform physical gelatin gels into chemical ones, hence chemically crosslinked hydrogels. Glutaraldehyde [5,6], polyphenols [7,8] and genipin have been used as crosslinking agents [9]. Composite materials based on the addition of hydroxyapatite also allow us to increase the stiffness of gelatin-based films [10]. All those crosslinking methods not only increased the mechanical properties but also markedly reduced the swelling behavior of the hydrogels. In the case of genipin, issued from gardenia fruits, approximately 86% of the amino groups in gelatin A underwent covalent modification in the presence of genipin at 2% (*w*/*v*), namely 20 mg/mL, and the endothermic peak of the differential microcalorimetry scans shifted from 91 to 98 °C when the genipin concentration increased from 0 to 20 mg/mL [9].

In addition, porcine pericardium crosslinked with genipin was more stable upon prolonged storage in phosphate-buffered saline than its glutaraldehyde counterpart and displayed negligible cytotoxicity [11]. Genipin allows us to crosslink gelatin through amino groups belonging, for instance, to L-Lysine residues in the presence of dissolved oxygen. It has been shown that Young’s modulus of dry gelatin A films, as measured by tensile tests, increases by a factor of 6–7 to reach a plateau at approximately 6.8 ± 0.9 MPa when the genipin concentration is increased to its limit of solubility in water, namely 20 mg/mL [9].

The aim of the present investigation was to measure the elastic modulus of gelatin B gels (at a constant gelatin concentration of 10% *w*/*v*) as a function of the genipin concentration directly added to the hot gelatin sol before the onset of gelation but using a local probe to investigate the homogeneity of the mechanical properties at the scale of the probe used in the ferrule-top micro-indentation experiment [12,13,14], namely a few µm. This indentation method is close to the colloidal probe AFM but with a cantilever deflection detection method based on interferometry [12,13]. Among other examples, ferrule-top micro-indentation was used to map the viscoelastic properties of brain tissue [14] and the heterogeneous elastic moduli of chicken embryos [15]. The same technique was also used to characterize elasticity gradients produced by the enzymatically controlled dephosphorylation of Fmoc-FF-phosphorylatedY peptide leading to its self-assembly and formation of stiff structures along the surface of hydroxypropyl(methylcellulose) hydrogels. Those elasticity changes were correlated with changes in chemical composition [16]. In the present investigation, the data obtained from ferrule-top micro-indentation was be compared with macroscopic measurements using a shear rheometer as a function of the genipin concentration used to crosslink the gelatin hydrogels. The investigated gels were called gelatin–genipin *x* gels, where *x* is the genipin concentration (in mg/mL) used to crosslink the gels.

## 2. Results and Discussion

### 2.1. Reaction Kinetics between Gelatin and Genipin

**Figure 1:**
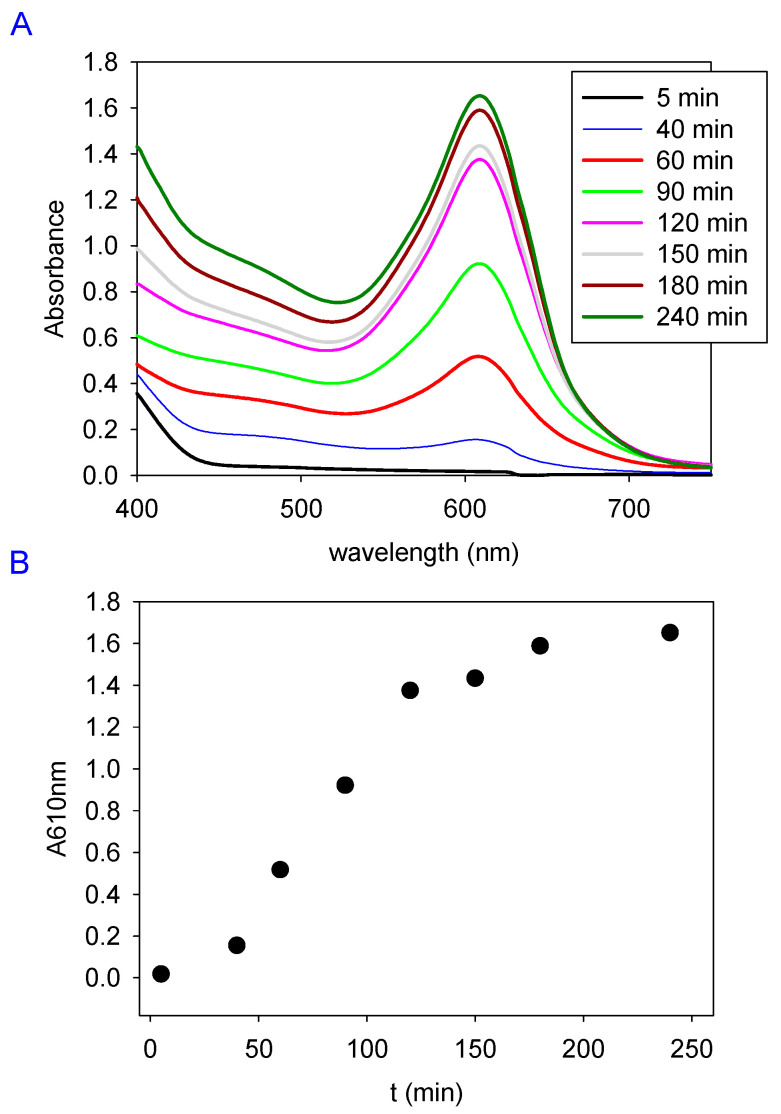
(**A**) Absorbance spectra of a gelatin–genipin 2 gel deposited on a quartz slide as a function of time as indicated in the inset. (**B**) Time evolution of the absorbance at 610 nm of the gelatin–genipin 2 gel deposited on a quartz slide.

The reaction occurring (at pH = 5.0 and 25.0) between gelatin (10% (*w*/*v*)) in the presence of genipin at 2 mg/mL and in the presence of air was investigated by means of UV-vis spectroscopy (Figure 1). It appears that the absorbance at 610 nm, corresponding to the peak position, was due to the blue color of the reaction product [11], increasing to a saturation value after approximately 2 h, implying the achievement of the reaction in this gel section was roughly 1 mm in thickness. Note that the monitoring of the crosslinking was performed with the gel, approximately 1 mm thick, deposited on a quartz plate to ensure a continuous flow of oxygen from the air. In a deaerated medium, the reaction between genipin and gelatin does not occur.

The reaction between gelatin and genipin was slightly faster in the presence of higher genipin concentrations.

### 2.2. Ferrule-Top Micro-Indentation Experiments

**Figure 2:**
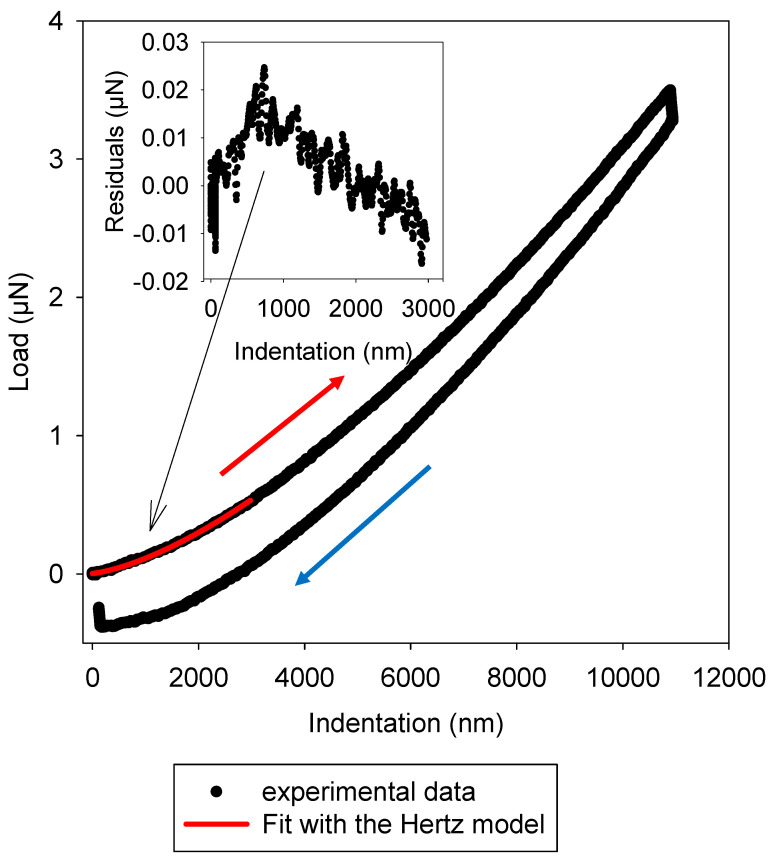
Prototypal indentation curve with the approach and retraction part of the cantilever on a gelatin-genipin 2 gel after 4 h of ageing. The red line corresponds to the fit of the Hertz model to the load-indentation curve during the approach phase. The red and blue arrows correspond to the approach and retraction regime, respectively.

The gelatin–genipin *x* gels were poured into glass Petri dishes to cover approximately half of their bottom surface as described in the Section 4. They were then investigated by ferrule-top micro-indentation after 1 h of gelation at ambient air before being recovered with sodium acetate buffer. After that, indentation experiments were performed as a function of time at different locations around the region of interest, corresponding to domains where the gel thickness was higher than one millimeter. A typical approach retraction curve is displayed in Figure 2. In all our experiments, independent of the genipin concentration, indentation was performed in 2 s at 5 µm/s, with a maximal indentation depth of 700 nm; the probe was left in contact with the gel for 4 s at a constant indentation depth and removed after 2 s at a speed of 5 µm/s. The Hertz model was then used to fit the load versus indentation part of the curve up to 15% of the maximal load (red curve in Figure 2). For such soft materials, the Hertz model was favored with respect to other mechanical models, for instance, the model by Oliver and Pharr, to fit the data [17]. In all our experiments, the quality of the Hertz fit was excellent, with residuals between the fitted values and the measured values distributed in a Gaussian manner around zero (inset in Figure 2). However, around 10% of the indentation curves could not be used because of the absence of contact between the probe and the gelatin–genipin *x* gel.

All the indentation curves shown in Figure 2 display hysteresis between the approach and the retraction regime. In addition, during the 4 s of contact between the bead and the gel, the load decreased, a sign of stress relaxation. Such behavior is characteristic of viscoelastic materials [17]. The stress relaxation was however less pronounced as the genipin concentration used to crosslink the gels increased. In addition, the bead adhered to the gels during the retraction phase in a genipin concentration-dependent manner. Such an adhesion event is clearly visible in Figure 2. The interpretation of such data requires a good estimation of the contact area between the indented bead and the hydrogel. This surface area is dependent on the maximal indentation depth and the bead radius. These aspects will be addressed in a forthcoming article.

**Figure 3:**
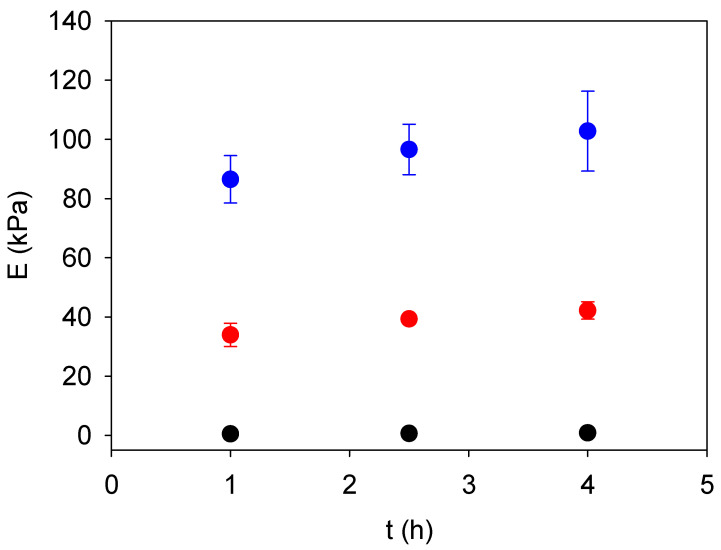
Time evolution of Young’s modulus of gelatin–genipin *x* gels with *x* = 0 (black disks, ●), *x* = 5 (red disks, ●) and *x* = 10 (blue disks, ●) mg/mL. The data correspond to 25 individual measurements performed at different locations on the gel surface. The error bars correspond to ± one standard deviation.

Young’s modulus of the gelatin–genipin *x* gels was measured as a function of the ageing time and for different values of the genipin concentration (Figure 3). It was determined that only a slight increase in stiffness was found between 1 and 4 h of genipin-induced crosslinking. These findings confirm the fast effect of genipin on the reaction with gelatin under the experimental conditions used here, namely at pH = 5.0 (Figure 1).

**Figure 4:**
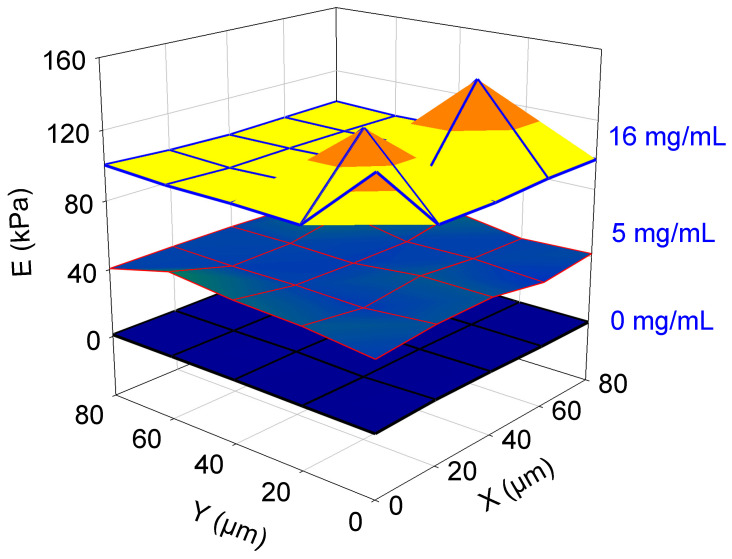
Maps (80 µm × 80 µm) of Young’s modulus of gelatin–genipin *x* gels as a function of increasing genipin concentrations (as indicated on the right vertical scale) after 4 h of gelation in the absence or the presence of genipin. Each vertex on those maps corresponds to a local determination of Young’s modulus.

In the following, we will nevertheless concentrate on Young’s modulus (and rheological behavior of gelatin–genipin *x* gels) after 4 h of crosslinking. Figure 4 shows that Young’s moduli of those gels exhibit a pretty homogeneous distribution over 80 × 80 µm² surface areas up to the maximal investigated genipin concentration of 16 mg/mL. However, at the highest genipin concentrations, the distribution of Young’s moduli over the surface becomes less homogeneous, suggesting that the crosslinking reaction may induce some domains which are stiffer than others. This may be related to the limited solubility of genipin in water at such high concentrations. These data acquired by ferrule-top micro-indentation provide information regarding the local value of Young’s modulus at a resolution of approximately 10 µm (herein, we used a bead 26 µm in radius glued at the extremity of the cantilever), which is absolutely not accessible with mechanical characterization methods at the macroscale such as rheology. It was the major aim of this article to demonstrate the homogeneity of the Young’s modulus of the gelatin surface after crosslinking with genipin. This aim was reached but in a somewhat less satisfactory manner at the highest genipin concentration used.

### 2.3. Characterization by Rheology

**Figure 5:**
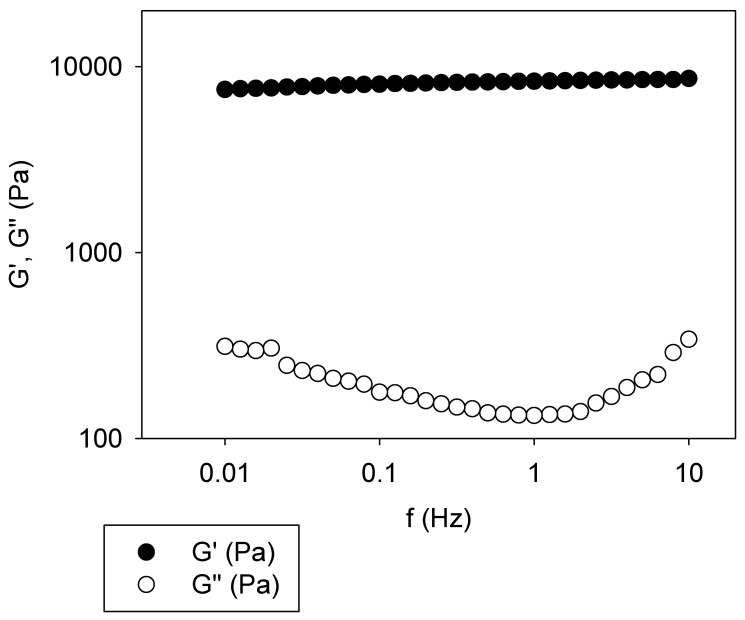
Frequency sweep experiment performed on gelatin–genipin 3 gel after 4 h of crosslinking.

For the sake of comparison with ferrule-top micro-indentation measurements, the shear modulus of the same gels was measured during frequency sweep experiments by means of rheology after 4 h of gelatin–genipin reaction. Note that the gelation kinetics could not be followed by means of shear rheology because of the non-uniform air supply between the two stainless steel plates of the rheometer. Indeed, in such preliminary experiments, only the outer perimeter of the gels was colored blue, a sign of the gelatin–genipin reaction (Figure 1), whereas the central part of the gel was much less colored. Therefore, experiments as those shown in Figure 5 were performed with gels formed in a Petri dish for 4 h and deposited on the rheometer lower plate before contact was made with the upper plate (see Materials and Methods).

In all experiments, even for uncrosslinked gelatin, the storage modulus was almost independent of the frequency between 0.01 and 10 Hz. These average plateau values will be used in Section 2.4 for comparison with Young’s moduli obtained by ferrule-top micro-indentation.

### 2.4. Influence of the Genipin Concentration on the Mechanical Properties of Gelatin Gels

**Figure 6:**
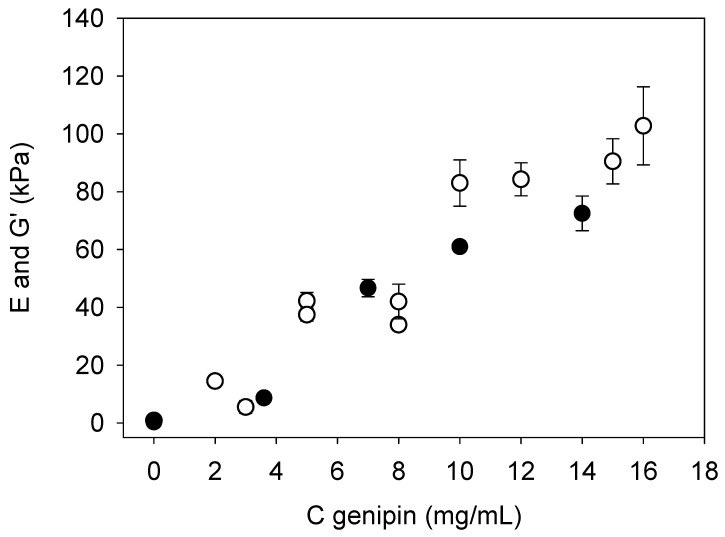
Evolution of the Young’s (◯) and storage moduli (●) of gelatin–genipin *x* gels after 4 h of reticulation in the presence of *x* mg/mL genipin in the gelling medium.

The Young’s moduli and the storage moduli of the gelatin–genipin *x* gels and after 4 h of reaction are combined in Figure 6. Good correlation between both mechanical characterization methods was found. The Young’s moduli and the storage moduli (obtained from the plateau values in curves such as those displayed in Figure 5) are of the same order of magnitude, whatever the genipin concentration. Since the nature of the applied stress is not the same (compression and shear for ferrule-top micro-indentation and rheology, respectively), both values of the moduli cannot be the same, but they should be of the same order of magnitude being related through the Poisson ratio of the investigated material. This was indeed the case (Figure 6).

### 2.5. Discussion

Herein, we have shown that an increase in Young’s modulus and the storage modulus by two orders of magnitude can be obtained by increasing the genipin concentration up to 12–16 mg/mL and seems to reach a plateau at those concentrations (Figure 6). The obtention of a plateau value in the elastic modulus is in agreement with previous reports [9]. However, in the article by Bigi et al., Young’s modulus of type A gelatin (from 5% *w*/*v* solutions) was found to increase only by a factor of 6–7 when the genipin concentration was increased from 0 to 15 mg/mL, with however much higher values (in the MPa range) than in the present investigation [9]. Their higher values can be easily explained, considering that their experiments, tensile tests, were performed in the dry state. The much lower influence of the genipin concentration on Young’s modulus may be due to the fact that Bigi et al. prepared their crosslinked hydrogels in a different manner than in the present investigation. Indeed, Bigi et al. allowed for the gelatin film to dry before being put in contact with a genipin solution at the desired concentration; crosslinking was then performed at 24 h (pH = 7.4) and at room temperature [9]. In the present investigation, genipin was added to the hot gelatin solution, at the desired concentration, before cooling down to 25 °C for a maximum of 4 h.

The most interesting finding of the present investigation is that Young’s moduli of the gelatin–genipin *x* gels were measured at a microscopic scale using ferrule-top micro-indentation and that the obtained values correlate well with the storage moduli obtained at a macroscopic scale by shear rheology. The observation of some local variations in the local Young’s modulus after crosslinking with the highest genipin concentration (for instance, at 16 mg/mL, see Figure 5) may not be of major importance for biological applications. Indeed, for most of those applications, a Young modulus higher than 1 kPa (the modulus of the gelatin B gels used herein) is required, but values higher than 100 kPa in the hydrated state (the average value reached between 12 and 16 mg/mL in genipin) are often not required. Gelatin–genipin gels of higher Young’s moduli may find some applications in packaging.

The limited solubility of genipin above 12–14 mg/mL in the gel state is observed through some increase in the gels’ turbidity. Such limited solubility may lead to the formation of genipin-based nanoprecipitates for which only a number of genipin molecules are available to react with the active amino acids on gelatin. In addition, at the locations of those nanoprecipitates, the gel behaves locally as a composite material which could display a higher value of Young’s modulus.

## 3. Conclusions

The protocol used to crosslink gelatin gels by directly adding genipin (dissolved in ethanol) in hot gelatin B solution before the onset of gelation allows us to increase Young’s modulus by two orders of magnitude with respect to uncrosslinked gel. This increase is much more pronounced than in the situation where gelatin films are crosslinked in a post-gelation manner. In addition, the data obtained by ferrule-top micro-indentation show a good homogeneity of the local Young’s modulus, measured with a lateral resolution of approximately 10 µm, with some more pronounced deviations at the highest used genipin concentration (that may induce some heterogeneous changes in the crosslink density, the genipin concentration being close to its solubility limit). In addition, the local ferrule-top measurements correlate semi-quantitatively with the storage moduli obtained by rheometry.

## 4. Materials and Methods

### 4.1. Chemicals

Gelatin from bovine skin (G6650, Type B) was purchased from Sigma-Aldrich (St. Louis, MO, USA) and used without further purification. Genipin (ref 078-03021) was provided from Wako (Neuss, Germany). All the gelatin solutions were developed in 50 mM sodium acetate buffer (ref. 6268, Merck, Darmstadt, Germany) and the pH adjusted to 5.0 with concentrated hydrochloric acid (ref. 403871, Carlo Erba, Cornaredo, Italy). The pH was monitored using a calibrated pH meter (SevenCompact, Mettler-Toledo, Columbus, OH, USA). Genipin was dissolved in absolute ethanol (ref. 524125, Carlo Erba) before being mixed with aqueous gelatin solutions. This allowed us to solubilize genipin up to 200 mg/mL in ethanol to reach a final concentration of up to 16 mg/mL in the volume of the gel.

### 4.2. Gel Preparation

In total, 1.25 g of gelatin were dissolved in 10.0 mL sodium acetate buffer at 70 °C under magnetic stirring (400 rpm). To this solution, 2.5 mL of an ethanolic solution of genipin was added. Note that genipin was dissolved in absolute ethanol to improve its solubilization. The amount of genipin was changed to vary its final concentration between 0 (control experiment) and 16 mg/mL in the final gelatin + genipin mixture. At the first level of approximation, we neglected the small volume change occurring upon mixing the aqueous gelatin solution and the ethanolic genipin solution. After 2 min of homogenization, this mixture was poured into a clean glass Petri dish to allow for gelation at room temperature (25 ± 1) °C. The gel occupied only half of the surface area of the Petri dish in the case of gel characterization by means of ferrule-top micro-indentation to allow for internal calibration of the used probe on the uncovered glass substrate. After 1 h of gelation, the gel and the uncovered part of the Petri dish were recovered with sodium acetate buffer. The time evolution of Young’s modulus of the gel was then investigated with ferrule-top indentation. In the case of rheological characterization, the whole surface area of the Petri dish was filled with the gelatin + genipin *x* hot mixture. Gelation was allowed for the time necessary to reach a steady value of Young’s modulus as determined previously by ferrule-top indentation. After that time, the gel was detached from the Petri dish, and its thickness was determined with a caliper before deposition on the stainless steel plate of the rheometer, conditioned at (25.0 ± 0.1) °C.

### 4.3. Ferrule-Top Micro-Indentation Measurements

The gelatin–genipin *x* gels were aged at least 1 h in the containing Petri dish before being put in contact with sodium acetate buffer. Their stiffness was then measured by ferrule-top indentation using a Chiaro device (Optics 11, Amsterdam, The Netherlands). The used cantilever had a spring constant of 4.34 N/m (as provided by the furnisher) and was fitted with a glass bead 26 µm in radius at its extremity. The cantilever was covered with a thin gold layer at its extremity, allowing us to follow its position as compared to the position of the moving piezoelectric crystal onto which it was fixed by means of interferometric detection [12,13]. Between two successive experiments, the glass bead was immersed for at least one hour in hot distilled water (50 °C or above) to desorb the eventually adsorbed gelatin material. Before each experiment, the glass bead was intensively washed with isopropanol and distilled water. Experiments started with optical calibration to check if the cantilever motion could indeed be recorded interferometrically [12]. The bead was then put in contact with, not covered by, the hard gel-part of the Petri dish in order to determine the ratio between the displacement imposed by the piezoelectric crystal and the motion of the cantilever (recorded by the interferometer). The ratio between both displacements, different from 1 due to the viscous drag force undergone by the cantilever, constitutes the geometric factor required to record the load-indentation curve of the investigated soft material. The indentation depth was restricted to 700 nm in all the experiments, which constitutes only 3% of the bead radius and much less concerning the ratio between the maximal indentation and the thickness of the gel (more than 1 mm). In these conditions, the estimated gel stiffness should not be influenced by the presence of the hard glass substrate. In each experiment, the contact between the probe, the glass bead, and the surface of the gelatin–genipin *x* gel was found by approaching the probe to the gel’s surface at a constant speed of 5 µm/s in order to reach a threshold force of 50 nN. Then, the mapping of the surface stiffness started by performing 25 successive approach–retraction curves. Indentation was performed in 2 s, and the probe was left in contact with the gel at a constant indentation for 4 s before retraction for 2 s to reach a load equal to zero. The retraction curve allows us to estimate the adhesion between the bead and the surface of the gel, whereas the contact time between the bead and the gels allows us to estimate the stress–relaxation, a characteristic of a viscoelastic material. However, in the present investigation, the focus was put exclusively on the approach curves. These curves allow us to estimate the apparent Young’s modulus of the gelatin–genipin *x* gels. To that aim, we used the Hertz model as recommended for soft materials [17].

### 4.4. Rheology

The frequency sweep experiments were performed on planar gelatin–genipin *x* gels with an Axis Ultra rheometer from Malvern Instruments (UK). The position of the upper planar stainless steel plate was adjusted to a position allowing for a small compression of the gel (whose thickness was previously measured with a caliper). The gelation kinetics of the formulated gelatin–genipin *x* mixtures could not be accurately investigated, as explained in Section 2. Frequency sweep measurements were performed at a constant strain of 1% between 10 and 0.01 Hz at (25.0 ± 0.1) °C.

### 4.5. UV-Vis Spectroscopy

The kinetics of the reaction between gelatin and genipin were followed by means of UV-vis spectroscopy (UV-mc² spectrophotometer, Safas, Monaco) between 400 and 750 nm to evaluate the time evolution of the resulting blue coloration. Since this reaction depends on an oxygen flow, it could not be investigated in a regular cuvette because of oxygen diffusion limitations, but 1.0 mL of a freshly prepared gelatin–genipin *x* mixture was spread on a freshly cleaned quartz slide (Thuet, Blodelsheim, France) and allowed for gelation, which occurred in less than 5 min. The spectrum of the gel was then regularly measured against a reference quartz plate coated with an unmodified gelatin gel.

## Data Availability

The additional curves, from ferrule-top micro-indentation and rheology can be available on request. The data presented in this study are available on request from the corresponding author.
